# GSK3 Inhibitor-Induced Dentinogenesis Using a Hydrogel

**DOI:** 10.1177/00220345211020652

**Published:** 2021-06-21

**Authors:** A. Alaohali, C. Salzlechner, L.K. Zaugg, F. Suzano, A. Martinez, E. Gentleman, P.T. Sharpe

**Affiliations:** 1 Centre for Craniofacial and Regenerative Biology, Faculty of Dentistry, Oral & Craniofacial Sciences, King’s College London, London, UK; 2 Department of Dental and Oral Health, Prince Sultan Military College of Health Sciences, Dammam, Saudi Arabia; 3 Department of Reconstructive Dentistry, University Center for Dental Medicine, University of Basel, Basel, Switzerland; 4 Centro de Investigaciones Biologicas–CSIC, Madrid, Spain

**Keywords:** tissue engineering, mineralization, Wnt signaling, dental pulp, dentine, stem cells

## Abstract

Small-molecule drugs targeting glycogen synthase kinase 3 (GSK3) as inhibitors of the protein kinase activity are able to stimulate reparative dentine formation. To develop this approach into a viable clinical treatment for exposed pulp lesions, we synthesized a novel, small-molecule noncompetitive adenosine triphosphate (ATP) drug that can be incorporated into a biodegradable hydrogel for placement by syringe into the tooth. This new drug, named NP928, belongs to the thiadiazolidinone (TDZD) family and has equivalent activity to similar drugs of this family such as tideglusib. However, NP928 is more water soluble than other TDZD drugs, making it more suitable for direct delivery into pulp lesions. We have previously reported that biodegradable marine collagen sponges can successfully deliver TDZD drugs to pulp lesions, but this involves in-theater preparation of the material, which is not ideal in a clinical context. To improve surgical handling and delivery, here we incorporated NP928 into a specifically tailored hydrogel that can be placed by syringe into a damaged tooth. This hydrogel is based on biodegradable hyaluronic acid and can be gelled in situ upon dental blue light exposure, similarly to other common dental materials. NP928 released from hyaluronic acid–based hydrogels upregulated Wnt/β-catenin activity in pulp stem cells and fostered reparative dentine formation compared to marine collagen sponges delivering equivalent concentrations of NP928. This drug-hydrogel combination has the potential to be rapidly developed into a therapeutic procedure that is amenable to general dental practice.

## Introduction

Caries remains a significant clinical problem in dentistry where basic treatment protocols are rooted in the use of inorganic materials to replace mineralized tissue removed during treatment. Dentine, which constitutes the bulk of the tooth mineralized tissue, is capable of a considerable level of regeneration following damage (Smith 2002; Yu et al. 2015; Ricucci et al. 2019). Odontoblast destruction and pulp exposure lead to activation of resident stem cells that differentiate into odontoblast-like cells to produce reparative dentine (dentine bridge) (Feng et al. 2011; Kaukua et al. 2014; Babb et al. 2017). This stem cell activation is dependent on Wnt/β-catenin signaling, which is upregulated following tissue damage, and the level of reparative dentine produced is directly related to the level of signaling activity (Clevers and Nusse 2012; Hunter et al. 2015; Babb et al. 2017; Neves et al. 2017). Based on these observations, we developed a small molecule–based approach to overactivate Wnt/β-catenin in tooth cavities as a way of enhancing reparative dentine formation. Numerous small-molecule drugs that inhibit glycogen synthase kinase (GSK), a key intracellular kinase and negative regulator of the pathway, have been developed (Meijer et al. 2004; Sato et al. 2004). In both dental pulp cell cultures and in vivo mouse and rat molar damage models, we observed rapid upregulation of Wnt/β-catenin signaling from both adenosine triphosphate (ATP)–competitive and ATP-noncompetitive classes of GSK3 inhibitor drugs (Neves et al. 2017; Zaugg et al. 2020). In vivo, the target cells of the drugs are stimulated to proliferate and differentiate into odontoblast-like cells that produce reparative dentine (Neves et al. 2017). The reparative dentine produced in mice and rat molar cavities completely fills the lesion and has a mineral content and composition similar to that of normal dentine (Neves et al. 2017; Zaugg et al. 2020). In these models, therefore, addition of exogenous cavity-filling materials such as calcium hydroxide or mineral trioxide aggregate (MTA) is not required, as the cavities self-fill with reparative dentine.

Many common GSK3 inhibitors are poorly water soluble, requiring the addition of up to 20% dimethyl sulfoxide (DMSO) for delivery. For localized application of these drugs into tooth cavities, an aqueous formulation is preferred. We have previously used a simple, clinically approved marine collagen sponge to deliver thiadiazolidinone (TDZD) drugs. The sponges release the drug rapidly and biodegrade as reparative dentine forms; however, clinical handling of the sponges is not ideal since they require individual placement in the cavity with forceps.

**Figure 1.**
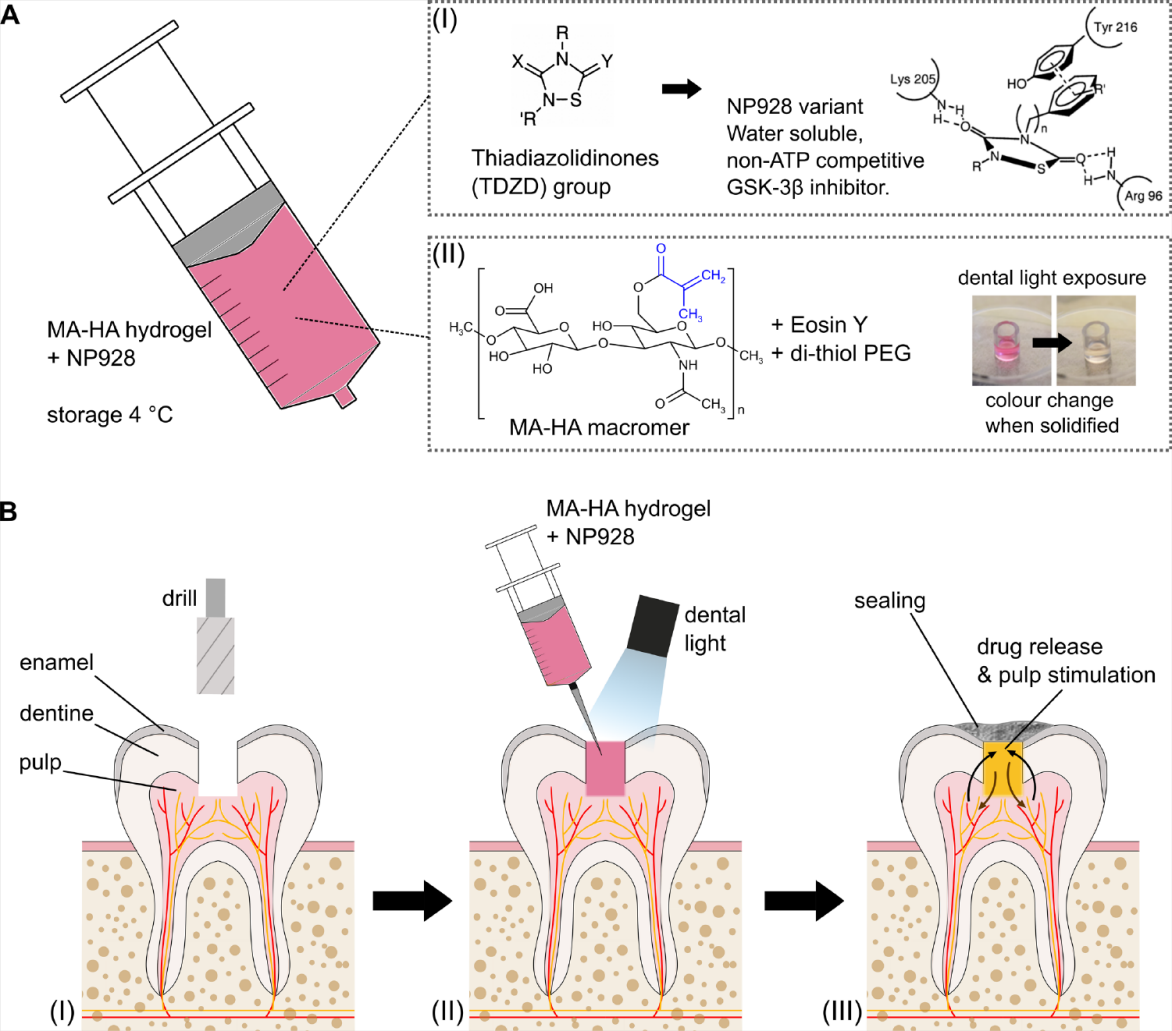
Drug delivery system for clinical application. (**A**) Methacrylate (MA)–hyaluronic acid (HA) hydrogel precursor solution containing NP928 can be stored at 4°C before use. (**A-I**) NP928 is part of the thiadiazolidinone (TDZD) family (left), modified for increased water solubility. It can activate the Wnt/β-catenin pathway by binding to glycogen synthase kinase 3 (GSK3), without competing with adenosine triphosphate (right; proposed TDZD/GSK3 binding action). (**A-II**) Hydrogel precursor solution contains 1% (w/v) MA-HA macromers, as well as the photoinitiator Eosin Y and dithiol-PEG. Upon blue light exposure, Eosin Y deprotonates dithiol-PEG, which can then bind to the methacrylate groups (MA) of the HA, leading to hydrogel gelation. This reduces Eosin Y from a red to a yellow color, which indicates gel formation. (**B**) Clinical application. (**B-I**) Removal of affected enamel and dentine tissue. (**B-II**) MA-HA hydrogel precursor solution containing NP928 is applied by syringe. Due to its low viscosity, the precursor solution can be easily removed if necessary. Dental light exposure (400–500 nm wavelength) solidifies the material within a few minutes. (**B-III**) A sealing was applied to protect the cavity during dentinogenesis. The underlying hydrogel releases NP928, stimulating Wnt/β-catenin activity in pulp cells. As the hydrogel degrades, reparative dentine is formed.

Here, we describe a new GSK3 inhibitor small-molecule drug, NP928, that has increased aqueous solubility compared to other TDZD drugs (Fig. 1A-I) and can activate Wnt/β-catenin pathway similarly to tideglusib. To deliver this drug, we used a hyaluronic acid–based hydrogel (Fig. 1A-II), which (Salzlechner, Haghighi, et al. 2020; Salzlechner, Walther, et al. 2020) allows for the incorporation of bioactive drugs and is suitable for direct placement by syringe in clinical contexts (Fig. 1B-I and B-II). Once placed in the defect, the liquid solidifies upon exposure to a visible light source widely available in dental clinics. After solidification, the hydrogel allows for the application of a bacterial tight seal (capping material) to protect the tooth cavity (Fig. 1B-III). The hydrogel diffusivity allows rapid drug release kinetics without tempering its bioactivity and safely degrades, permitting the formation of new dentine. Our approach demonstrates that NP928 combined with the hydrogel effectively stimulates reparative dentine formation in vivo and can be easily deployed in a clinical context.

## Materials and Methods

### NP928 Synthesis

The small thiadiazolidinone molecule NP928 was synthesized following a 1-step convergent pathway, as previously described (Martinez et al. 2002; Medina Padilla et al. 2013). Its main metabolite, *N-*benzyl-*N*′-ethyl-urea, was also synthesized according to a previously reported method using a 1-step synthesis (Martinez et al. 2006).

### Cytotoxicity Assay

17IA4 mouse dental pulp cells were cultured in 96-well plates (0.32 × 10^5^) in αMEM/L-glutamine with 15% (v/v) fetal bovine serum (FBS) and 1% (v/v) antibiotic-antimycotic (ABAM) (15240062; Thermo Fisher Scientific). Cells were incubated at 37°C, 5% CO_2_/95% air, and 100% humidity for 24 h. NP928 was dissolved in DMSO at indicated concentrations. Media on 17IA4 cells were replaced with conditioned media (1 µL NP928 in DMSO + 99 µL media), 1% DMSO (1 µL DMSO + 99 µL media), or control media (100 µL media alone) and incubated for 24 h. Cell metabolic activity was assessed by adding 20 µL of a 5-mg/mL solution of MTT (3-(4,5-dimethylthiazol-2yl)-2,5-diphenyltetrazolium bromide, M2128; Sigma) in phosphate-buffered saline (PBS) to each well, prior to incubation for 4 h. The solution was aspirated and 200 µL DMSO added. Absorbance was measured on a colorimetric plate reader (CLARIOstar Plus; BMG LABTECH) at 450 nm with background subtraction at 630 nm.

### In Vitro Drug Release

17IA4 mouse dental pulp cells were plated in 24-well plates (0.5 × 10^6^) for 24 h prior to drug treatment. Media were replaced with conditioned media consisting of NP928 at concentrations of 500 nM and 100 nM (1% v/v DMSO/media), DMSO (1% v/v DMSO/media), or media alone. After 24 h, the cells were washed with PBS and collected using Buffer RLT Plus (74034; Qiagen). RNA was extracted using RNeasy Plus Micro Kit (74034; Qiagen).

### Drug Released from Hydrogel

Hyaluronic acid (HA) sodium salt (100–150 kDa; Lifecore Biomedical) was modified with methacrylate (MA) groups using an aqueous synthesis (Salzlechner, Haghighi, et al. 2020). The resulting monomer was lyophilized and sterilized with 54-kGy gamma irradiation. ^1^H NMR analysis confirmed successful modification of the HA with a total degree of modification of 19%. The lyophilized MA-HA monomer (1% w/v) was dissolved in 65 mM dithiol-PEG (1 kDa; JenKem Technology) and 154 μM Eosin Y with NP928, DMSO, or cell culture media alone. Then, 20 µL of solution was solidified by exposure to blue light (400–500 nm, 500–600 mW·cm^−2^, 4 min) using an OmniCure light system (S1500, Lumen; Excelitas Technologies). The resulting hydrogels were placed in 2 mL of culture media, resulting in a 1% concentration of the hydrogel in the media and incubated to allow NP928 to leach into the media. Supernatant were collected after 5 min, 30 min, 2 h, and 6 h and stored at −20°C. Mouse dental pulp cells (17IA4) were plated in 24-well plates (0.5 × 10^6^) using growth media (with 10% FBS and 1% ABAM) and incubated overnight. Growth media were replaced with conditioned media and incubated for 24 h. Cells were washed with PBS and collected using Buffer RLT Plus. RNA was extracted using the RNeasy Plus Micro Kit (74034; Qiagen). Quantitative polymerase chain reaction (qPCR) analysis was performed to quantify upregulation of the target gene by drug.

### QPCR

The extracted RNA (1 µg) was added with 2 µL random primer (M-MLV Reverse Transcriptase Kit; Promega) to Eppendorf tubes in RNase-free water. The resulting solution was incubated for 5 min at 70°C and then cooled on ice for 5 min. Thereafter, 11 µL master mix was added and incubated for 60 min at 42°C. The master mix was composed of 5 µL 5× buffer, 1 µL Moloney Murine Leukemia Virus Reverse Transcriptase (mmLV RT), 1.25 µL PCR nucleotide mix, and 3.75 µL RNase-free water. After 60 min of incubation, the samples were diluted with nuclease-free water (1:5) on ice and quantified using NanoDrop (ThermoFisher).

Each well plate comprised 4.8 µL 40 µg complementary DNA (cDNA), 0.1 µL (100 µM) reverse primer, 0.1 µL (100 µM) forward primer, and 5 µL LightCycler 480 SYBR Green Master Hot Start mix (Roche) as previously described (Neves et al. 2017). β-actin was used as the housekeeping gene (forward: GGCTGTATTCCCCTCCATCG; reverse: CCAGTTG GTAACAATGCCATGT), and Axin2 was the readout for Wnt/β-catenin activity (forward: TGACTCTCCTTCCAGAT CCCA; reverse: TGCCCACACTAGGCTGACA). LightCycler 480 sealing foil was used to seal the plate, and qPCR was run in a LightCycler 480. LightCycler 480 software was used for quantitative analysis and to collect the data. Data were analyzed using ∆∆CT. GraphPad Prism (GraphPad Software) was used for graph plotting and statistical analysis.

### Mouse Molar Injury

All animals used in this study were handled in accordance with UK Home Office Regulations (project license 70/7866 and personal license ID4E60F01), which was approved by the KCL animal ethics committee and complies with ARRIVE (Animal Research: Reporting of In Vivo Experiments) guidelines.

Six-week-old CD1 wild-type mice were used. The mice were anesthetized intraperitoneally with a solution containing Hypnorm (fentanyl/fluanisone; VetaPharma Ltd.), sterile water, and Hypnovel (midazolam; Roche) in a 1:2:1 ratio, at a rate of 10 mL/kg. Molar damage was performed as previously described (Babb et al. 2019). Briefly, 2 upper first molars were drilled using a rounded carbide burr FG ¼ with a high-speed handpiece. The drilling was from the occlusal surface of the tooth to the deep layer of the dentine, and the pulp of the middle cusp was exposed using a 30-gauge needle. NP928 drug was dissolved in DMSO and diluted to final concentrations (100 nM and 500 nM), accounting for an equivalent ratio 1% v/v of DMSO/PBS. The exposed pulp was treated with 0.2 µL NP928 (100 nM or 500 nM) delivered by collagen sponge (Kolspon; Eucare Ltd.) or with NP928 in a MA-HA hydrogel precursor solution (100 nM and 500 nM). Lyophilized MA-HA monomer (1% w/v) was dissolved in a solution containing 65 mM dithiol-PEG (1 kDa; JenKem Technology) and 154 μM Eosin Y, with a final concentration of 1% v/v drug in the hydrogel. Then, 0.2 µL NP928/MA-HA hydrogel was applied in the exposed pulp and was solidified by exposure to blue light (45 s). 0.2 µL DMSO diluted in PBS (delivered by collagen sponge) or diluted in hydrogel (1% v/v) was used as control. After the test and control materials were applied in direct contact to the vital pulp tissue, the cavity was sealed with glass ionomer cement as previously described (Babb et al. 2017; Neves et al. 2017). Vetergesic (Buprenophine; Ceva) and Antisedan (atipamezole hydrochloride; Orion Pharma) were used as an analgesic after the surgery and injected at the rate of 0.3 mg/kg by intraperitoneal injection. CD1 mice were sacrificed 4 wk after surgery. A total of 18 CD1 mice (36 molars) were used.

### Histological Staining

Extracted teeth were fixed for 24 h in 4% PBS buffered paraformaldehyde and decalcified in 19% ethylenediaminetetraacetic acid (EDTA) in H_2_O for 4 wk before being immersed in 30% sucrose/PBS overnight at 4°C (Neves et al. 2017). Teeth were embedded in wax blocks and sectioned (8 µm) using a microtome (Leica) onto SuperfrostPlus glass slides (J1800AMNZ; Thermo Fisher Scientific). Sections were stained using Masson’s Trichrome solution.

### Micro–Computed Tomography Scanning and Mineral Analysis

Teeth were dissected after 4 wk and fixed with 4% (w/v) paraformaldehyde for 24 h at 4°C and then placed in PBS. Teeth were scanned using a Scanco µCT50 micro–computed tomography (microCT) scanner. MicroView software (Parallax Innovations) was used for visualization and analysis. Two-dimensional (2D) images were obtained from microCT cross sections to evaluate mineral formation. The advanced region-of-interest (ROI) spline function was set as a standard for all teeth (ROI X = 0.2 mm, Y = 0.4 mm, and Z = 0.2 mm). Standardized contrast settings were set to window/level values of 23,000/18,000. Auto threshold was selected and water was set at −1,000 HU and bone density (HA) at 5,343 HU. Mineral analysis was performed to assess the area under the damage site. The complete ROI filled with mineralized tissue equaled an amount of 0.0017 mg.

### Statistical Analysis

The data are presented as means and standard deviations. Data represent at least 3 independent experiments. A 1-way analysis of variance (ANOVA) followed by a post hoc Tukey test was used for statistical analysis (*P* < 0.05).

## Results

### Properties of NP928 and Biological Activity

Preparation of the thiadiazolidinone NP928 C_11_H_12_N_2_O_2_S was straightforward using commercial isocyanate and isothiocyanate, obtaining the compound in high yields as a translucid syrup (Medina Padilla et al. 2013). The primary metabolite of NP928 was identified as the corresponding *N,N*′-disubstituted urea derivative, *N*-benzyl-*N*′-ethyl-urea. NP928 has reduced hydrophobicity compared to the previously studied molecule, tideglusib (C_19_H_14_N_2_O_2_S), but it retains the active site and activity of both molecules, as assayed by expression of Axin2 in dental pulp cells in vitro (Martinez et al. 2002; Babb et al. 2017; Neves et al. 2017).

**Figure 2.**
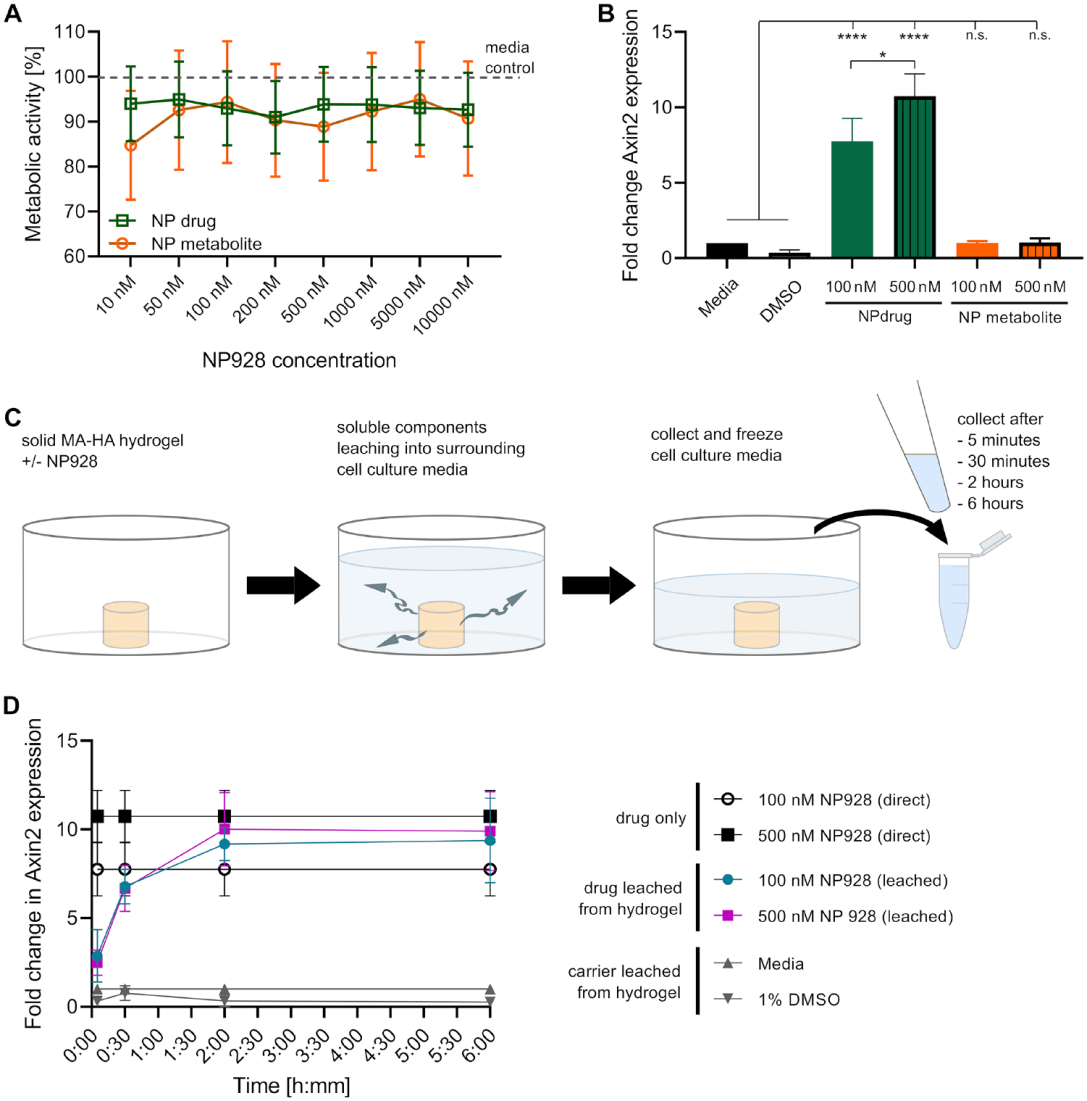
Drug toxicity, release, and bioactivity. (**A**) Metabolic activity of 17IA4 mouse dental pulp cells was not affected by NP928 or its metabolite when exposed to concentrations up to 10 μM for 24 h. (**B**) Axin2 expression of 17IA4 mouse dental pulp was significantly upregulated after treatment with both 100 nM and 500 nM NP928 (****P < 0.0001; 100 nM versus 500 nM; *P = 0.0122; one-way analysis of variance [ANOVA] with post hoc Tukey test). There was no effect on *Axin2* expression of the drug’s metabolite. (**C**) Conditioned media were created by soaking methacrylate (MA)–hyaluronic acid (HA) hydrogels without addition of drug, with drug carrier (1% DMSO), or with 100 nM and 500 nM NP928 at 37°C in a cell culture incubator for 5 min, 30 min, 2 h, or 6 h. (**D**) Mouse dental pulp cells (17IA4) were incubated for 24 h with the conditioned media (C). Quantitative polymerase chain reaction showed upregulation of *Axin2* expression from media exposed to an NP928-loaded hydrogel for 5 min and plateaued after a 2-h exposure. After 6 h, 100 nM NP928 leached from hydrogels reached an upregulation comparable to the direct drug application (*P* = 0.8000 with similar upregulation at 500 nM NP982; one-way ANOVA with post hoc Tukey test).

We then carried out in vitro experiments to determine the cytocompatibility of NP928 and its metabolite, as well as to assess its ability to regulate *Axin2* expression. Direct exposure experiments did not result in any significant toxicity from either the drug or its metabolite (Fig. 2A). Metabolic activity levels in cells exposed to the compounds at concentrations ranging from 10 nM to 10 μM were not different from that of media-only controls, and so concentrations of 100 nM and 500 nM were chosen for further investigation. We next assessed the ability of NP928 and its metabolite to regulate *Axin2* expression. We found that both 100 nM and 500 nM NP928 significantly upregulated expression of *Axin2* (****P < 0.0001) (Fig. 2B) but that the NP928-metabolite did not have a statistically significant effect.

To ensure biological activity of NP928 upon release from MA-HA hydrogels, we placed NP928-loaded hydrogels in cell culture media for up to 6 h, allowing the drug to leach into the media (Fig. 2C). These conditioned media were placed on a 17IA4 mouse dental pulp/odontoblast progenitor cell line to investigate its ability to regulate *Axin2* expression levels (Fig. 2D) (Lacerda-Pinheiro et al. 2012). Dental pulp cells treated with cell culture medium exposed to the hydrogel for 30 min showed significantly elevated *Axin2* expression compared to controls (both concentrations ***P = 0.0004). *Axin2* expression levels plateaued in cells treated with media exposed to hydrogels for 2 h and were not significantly different after up to 6 h (100 nM, ***P = 0.0003, 500 nM, ***P = 0.0002). Cells treated with conditioned medium from hydrogels containing 100 nM NP928 (6 h) had levels of *Axin2* expression that were similar to cells treated with conditioned medium from hydrogels containing 500 nM NP928 (6 h) (*P* = 0.9983). Moreover, expression levels in cells treated with 500 nM NP928 from hydrogels (6 h) were the same as cells treated directly with 500 nM NP928 (*P* = 0.9852). We found no statistical significance between the impact of drug concentration on *Axin2* expression of 100 to 500 nM NP928 leached from hydrogels (*P* = 0.9983) or applied directly (*P* = 0.2641).

#### NP928/MA-HA stimulation of reparative dentine

We next tested the effect of NP928 delivered from MA-HA hydrogel in an in vivo model. We have previously shown that MA-HA precursors gel upon a <30-s exposure to blue light, forming hydrogels with a G′ of approximately 200 Pa. To reduce gelation time to limit the length of the surgery, we exposed the gels to blue light for 45 s (compared with 4 min for the in vitro experiments), which rheological measurements have previously shown to only have a marginal effect on hydrogel mechanical properties (Salzlechner, Haghighi, et al. 2020).

We evaluated the reparative potency and clinical usability of NP928-loaded MA-HA hydrogels in the pulp damage model in wild-type mice. We delivered identical concentrations of NP928 (100 nM and 500 nM) either via MA-HA hydrogel or collagen sponges. The NP928/MA-HA hydrogel formulation could be prepared before the surgical procedure and left in the syringe, ready for direct placement into the lesion (see Appendix Movie 1). This is in contrast to the collagen sponges, which require placement of sponge using forceps. Excess hydrogel material was wiped off or aspirated and reapplied. Upon exposure to conventional dental light, the hydrogel changed color to yellow, providing a visual indication of solidification.

**Figure 3.**
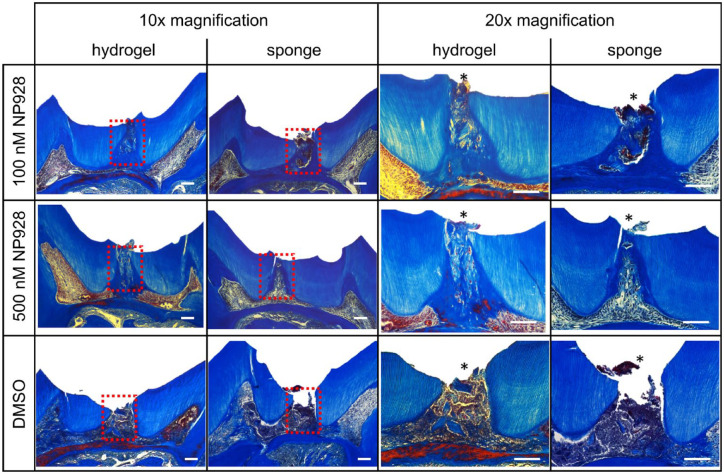
Histology of reparative dentine formation in CD1 wild-type mice. Molars were treated for 4 wk with NP928 released from both methacrylate (MA)–hyaluronic acid (HA) hydrogels and collagen sponges (100 nM and 500 nM) or DMSO (drug carrier). Decalcified teeth were sagittally sectioned and stained with Masson’s Trichrome solution. Pictures were taken with 10× and 20× magnification of the same sample. Asterisk indicates the drilling site. Scale bars = 100 µm.

Four weeks postdamage, histological sections through the molars revealed the extent of reparative dentine formation. Collagen sponge and MA-HA hydrogel alone (no NP928) produced little reparative dentine. The 100-nM and 500-nM concentrations of NP928 delivered with sponge or hydrogel both yielded more reparative dentine than the controls (Fig. 3).

**Figure 4.**
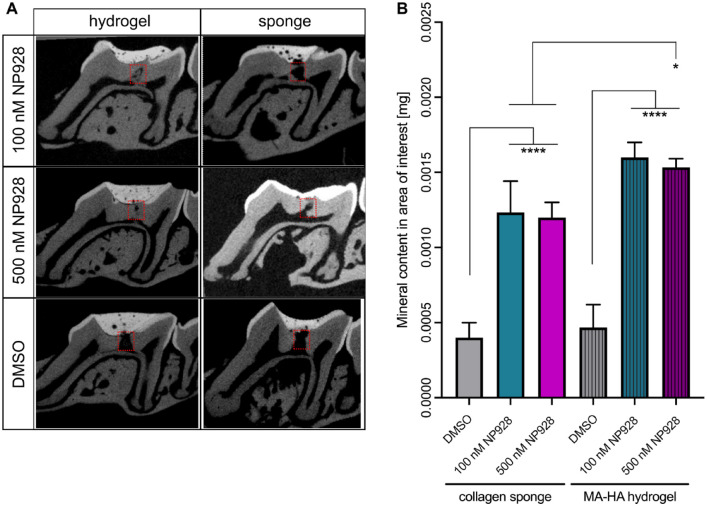
Qualitative and quantitative analysis of reparative dentine formation in CD1 wild-type mice. (**A**) Micro–computed tomography (CT) images of reparative dentin formation at the injury site after 4 wk treated with NP928 released from both methacrylate (MA)–hyaluronic acid (HA) hydrogel and collagen sponge (100 nM and 500 nM) or DMSO. Red squares indicate the area of the newly formed dentine under the drilling site. (**B**) Mineral formation analysis by microCT at the injury site after 4 wk treated with NP928 released from both MA-HA hydrogel and collagen sponge (100 nM and 500 nM) or DMSO. Images show the sagittal view of a first upper mouse molar. One-way analysis of variance with post hoc Tukey test.

MicroCT-based quantification of repaired molars showed teeth treated with MA-HA hydrogel precluded entry of the temporary capping into the lesion (Fig. 4A). Delivering 100 nM NP928 with sponges resulted in a statistically significant increase in reparative dentine compared to collagen sponge soaked with DMSO alone (****P < 0.0001 to sponge with DMSO) (Fig. 4B). Delivering 500 nM NP928 by collagen sponge similarly resulted in increased dentine volume (****P < 0.0001 to sponge with DMSO). The 100 nM or 500 nM NP928 from MA-HA hydrogel also led to a statistically significant increases in reparative dentine (****P < 0.0001 to hydrogel with DMSO). Overall, delivering NP928 via hydrogel resulted in 90% ± 3.5% (100 nM) and 94% ± 5.9% (500 nM) mineral content in the treated area. The mineral content in the repaired area of teeth treated with both 100 nM and 500 nM NP928 was similar (*P* = 0.9922). However, when we compared hydrogel to collagen sponge, hydrogel-mediated delivery resulted in more dentine formation, which was increased by 27.5% at a concentration of 100 nM (*P* = 0.0551) and 30% at 500 nM (*P = 0.0302).

## Discussion

Small-molecule drugs that stimulate Wnt/β-catenin have shown promise as a novel biological therapy for treating exposed pulpal lesions (Neves et al. 2017). In our previous studies, several different GSK3 inhibitor small-molecule drugs were capable of stimulating reparative dentine formation at low concentrations (Neves et al. 2017; Zaugg et al. 2020). This reparative dentine was biochemically indistinguishable from native dentine when analyzed by Raman spectroscopy (Zaugg et al. 2020). To move this therapy to first-in-human clinical trials, appropriate drug formulations that can deliver the drug locally into the tooth are required. One of these drugs, tideglusib, is particularly attractive since it has been shown to be safe when injected into patients at repeated high doses (del Ser et al. 2013; Tolosa et al. 2014; Lovestone et al. 2015). However, tideglusib has low aqueous solubility and in clinical trials is delivered in a granulate form suspended in water. However, even at DMSO concentrations applicable for the dental applications using low drug concentrations, the drug is delivered in 5% DMSO. Thus, we developed a modified version of tideglusib that removes the naphthyl moiety and increases solubility. This new drug, called NP928, is nontoxic and activates Wnt/β-catenin activity over a similar concentration range as tideglusib.

Clinical application of a drug to a tooth lesion requires a delivery method that is easy to use and compatible with existing dental equipment. Hydrogels are increasingly being developed for clinical applications that require drug or cell delivery, as they are biocompatible, and their properties can be tuned for specific applications (Foyt et al. 2018). We have previously reported on MA-HA hydrogels for maxillofacial applications (Salzlechner, Haghighi, et al. 2020; Salzlechner, Walther, et al. 2020). These materials are synthesized using an aqueous route, rendering them nontoxic (Renard et al. 1994). Moreover, they are inexpensive and easily manufactured from clinically approved materials. We designed the hydrogels so that they would be suitable for placement by syringe and could cross-link in situ using standard blue light sources, widely available in clinics. The MA-HA hydrogels are degradable by native hyaluronidases, which are ubiquitous in vivo. A biodegradable drug delivery system that uses a liquid that solidifies in tooth cavities and releases drug with the appropriate kinetics represents an advance over the surgical implantation of collagen sponges. In our previous collagen sponge–based model, quick release of NP928 allowed for rapid activation of Wnt/β-catenin signaling, fostering quick reparative dentine formation. It is possible to tune the properties of MA-HA hydrogels by increasing polymer concentration or the degree of chemical modification of the HA to slow drug release. However, here the 1% (w/v) MA-HA hydrogel formulation allowed for quick release, similar to that from the collagen sponge, driving activation of Wnt/β-catenin signaling and reparative dentine formation. In addition, hyaluronic acid may increase viability and survival of progenitor cells (Lisignoli et al. 2006; Bian et al. 2013; Ferreira et al. 2018). Significantly, we delivered NP928 into teeth in microliter volume solutions containing 1% (v/v) DMSO/PBS that could be reduced to zero in larger batch volumes of drug. In combination with the improved handling of this material, the hydrogel is superior to the sponge delivery, with an overall simpler user experience for the clinician. The hydrogel is also capable of delivering antimicrobials that might be used in conjunction with NP928 if necessary.

In conclusion, microdose concentrations of NP928 delivered into tooth cavities via a hydrogel can provide an effective, user-friendly, biologically based treatment for the restoration of deep caries lesions.

## Author Contributions

A. Alaohali, contributed to design, data acquisition, and analysis, drafted and critically revised the manuscript; C. Salzlechner, contributed to design and data acquisition, drafted and critically revised the manuscript; L.K. Zaugg, contributed to design, drafted and critically revised the manuscript; F. Suzano, contributed to data acquisition, drafted the manuscript; A. Martinez, contributed to conception and data acquisition, drafted the manuscript; E. Gentleman, P.T. Sharpe, contributed to conception, design, data analysis, and interpretation, drafted and critically revised the manuscript. All authors gave final approval and agree to be accountable for all aspects of the work.

## Supplementary Material

Supplementary material
